# Significant gender difference in serum levels of fibroblast growth factor 21 in Danish children and adolescents

**DOI:** 10.1186/1687-9856-2014-7

**Published:** 2014-05-23

**Authors:** Amalie Bisgaard, Kaspar Sørensen, Trine Holm Johannsen, Jørn Wulff Helge, Anna-Maria Andersson, Anders Juul

**Affiliations:** 1 Department of Growth and Reproduction, Rigshospitalet, Copenhagen University Hospital, section 5064 Blegdamsvej 9, DK-2100 Copenhagen, Denmark; 2 Faculty of Health and Medical Sciences, University of Copenhagen, Copenhagen, Denmark; 3 Department of Biomedical Sciences, Xlab, Center for Healthy Aging, University of Copenhagen, Copenhagen, Denmark

**Keywords:** Fibroblast growth factor 21, Metabolic syndrome, Oral glucose tolerance test

## Abstract

**Introduction:**

Fibroblast Growth Factor 21 (FGF21) is a novel metabolic factor with effect on glucose and lipid metabolism, and shown to be elevated in diseases related to metabolic syndrome. Due to the increasing frequency of metabolic syndrome in the pediatric population, and as FGF21 studies in children are limited, we investigated baseline serum levels of FGF21 in healthy children during an oral glucose tolerance test.

**Methods:**

A total of 179 children and adolescents from the COPENHAGEN Puberty Study were included. An OGTT with glucose and insulin measurements, a dual energy X-ray absorptiometry (DXA) scan and a clinical examination including pubertal staging were done on all subjects. Serum levels of FGF21, adiponectin, and leptin were determined by immunoassays at baseline.

**Results:**

The girls had significantly higher levels of FGF21 compared with boys (155 pg/mL vs. 105 pg/mL, *P* = 0.04). 38 children (21%) had levels below detection limit of assay. Baseline levels of FGF21 showed positive correlation with triglycerides, but no significant correlations were found between FGF21-concentration and body mass index (BMI), DXA-derived fat percentage, LDL- HDL- and non-HDL cholesterol, leptin or adiponectin levels, respectively. Neither was any correlation found between baseline FGF21-levels and the dynamic changes in glucose and insulin levels during the OGTT.

**Conclusion:**

FGF21 is independent of adiposity in children, and the significant metabolic effect seems to be limited to pathological conditions associated with insulin resistance. The higher levels of triglycerides in the girls may explain the significantly higher levels of FGF21 in girls compared with boys.

**Systematic review registration:**

The COPENHAGEN Puberty Study was registered in ClinicalTrials.gov (identifier NCT01411527), and approved by the local ethics committee (reference no. KF 01 282214 and KF 11 2006–2033).

## Introduction

Metabolic syndrome in children is becoming more frequent in the pediatric population, [1,2] and pathogenetic and prognostic factors are sought for. Recently, a new protein, fibroblast growth factor 21 (FGF21), has been suggested as a factor involved in regulation of carbohydrate and lipid metabolism [3-5].

FGF21 expression is induced by an increase in free fatty acids, and is regulated by peroxisome proliferator-activated receptor-alpha (PPAR-α) in the liver [6,7] and PPAR-gamma (PPAR-γ) in white adipose tissue [8,9]. In the liver, FGF21 acts as an endocrine factor, as it increases energy production and utilization of energy during prolonged fasting [10]. In white adipose tissue FGF21 acts as an autocrine factor, as it increases glucose uptake by up-regulating glucose transporter 1 (GLUT1) in the cell membrane [5]. Glucose also stimulates FGF21 expression through carbohydrate response-element binding protein (ChREBP) in the liver [11,12]. In adipocytes, it seems like PPAR-γ and ChREBP together can stimulate the expression of FGF21 [11]. Compared with insulin alone, glucose uptake into cultured adipocytes is enhanced by co-incubation with FGF21, suggesting that the effect of FGF21 is independent and additive to insulin. Accordingly, treatment with FGF21 to ob/ob mice and FGF21 transgenic mice over-expressing the human protein resulted in improved glucose clearance and insulin sensitivity during OGTT [5].

Diseases related to insulin resistance such as metabolic syndrome and type 2 diabetes mellitus have been related to increased levels of FGF21 [13-15]. In accordance, serum-concentrations of FGF21 correlated negatively with insulin sensitivity and positively with the hepatic insulin resistance index, HbA1c, fasting plasma glucose levels and two hour-plasma glucose levels after an oral glucose tolerance test in adult subjects, suggesting a relation with both hepatic and whole-body insulin resistance [13]. In children, knowledge on FGF21 and insulin resistance is limited. In a cohort study of both lean and obese children it was indicated that FGF21 levels were positively associated with free fatty acids, leptin and body mass index (BMI), respectively. This study further suggested that the increase in serum-concentrations of FGF21 in obese children was reversible with weight loss [16]. Giannini *et al.* confirmed this elevation of FGF21 in obese youth and in addition documented that FGF21 independent of visceral fat and insulin sensitivity correlated with fatty liver and markers of hepatic apopotosis [17].

The aim of this study was to evaluate the fasting concentrations of serum-FGF21 in children and adolescents in relation to anthropometrical measurements, pubertal stages, concentrations of lipids, leptin and adiponectin, and concentrations of glucose and insulin during a two-hour OGTT.

## Materials and methods

### Participants

Subjects were recruited as part of The COPENHAGEN Puberty Study [18,19] from primary schools in the Copenhagen community. A total of 179 healthy Caucasian children (114 girls) aged 8,5-16,1 years volunteered Table 1. Sixteen children and adolescents (8 girls) were clacified as overweight (BMI > 85th percentile for age) according to the CDC reference. None of the children meet the IDF crieteria for metabolic syndrome [20]. The cohort has previously been described in details [21-23]. In brief, all participants had pubertal stages evaluated according to Tanners classification. Twenty-seven of the girls were post-menarcheal. Total body fat and lean mass was evaluated with a whole-body dual-energy X-ray absorptiometry (DXA) scan using a CDR 1000/W densitometer (Hologic Inc., Bedford MA) with software version 6.2. Aerobic fitness was evaluated by assessing maximal oxygen uptake (V_O2_max) during a cycle ergometry test using an electronically braked cycle ergometer (Ergomedic 839: Monark, Varberg, Sweden). V_O2_max was measured directly using an online pulmonary gas analyzer system (Quark CPET; Cosmed, Rome, Italy).

**Table 1:** General metabolic characteristics related to metabolic syndrome in a group of 179 non-obese children (65 boys, 114 girls)

	**Boys**	**Girls**	**P-value**
	*n* = *65*	*n* = 114	
BMI (kg/m^2^)	18.3 (15.0-26.21)	18.0 (13.4-30.6)	0.21
Fat percentage (%)	18.1 (7.5-29.9)	21.1 (12.3-35.0)	< 0.001
Fasting glucose (mmol/L)	4.8 (3.9-5.9)	4.9 (3.2-6.7)	0.85
2-h glucose (mmol/L)	5.1 (2.8-7.3)	4.9 (2.9-8.3)	0.69
Fasting insulin (pmol/L)	43.0 (10.0-102.0)	48.0 (8.0-168.0)	0.03
Peak glucose (mmol/L)	7.0 (4.7-11.2)	7.4 (4.7-11.2)	0.41
Triglycerides (mmol/L)	0.6 (0.4-2.4)	0.7 (0.4-2.0)	0.001
HDL (mmol/L)	1.5 (0.9-2.1)	1.5 (0.7-2.2)	0.73
LDL (mmol/L)	2.1 (0.5-3.2)	2.2 (0.8-3.8)	0.29
Total cholesterol (mmol/L)	3.6 (2.4-4.6)	3.8 (2.4-5.6)	0.003
Non-HDL cholesterol (mmol/L)	2.0 (1.1-3.2)	2.4 (0.6-3.7)	0.001
Apolipoproten A1 (mmol/L)	50.6 (38.7-64.4)	51.7 (27.1-67.6)	0.40
Apolipoproten B (mmol/L)	2.1 (1.1-3.4)	2.2 (0.7-3.7)	0.06
Leptin (ng/mL)	3476.0 (1884.0-37065.0)	6353.0 (921-49495.0)	< 0.01
Adiponectin (μg/mL)	22876.5 (977.0-57085.0)	27757.5 (9375.0-60190.0)	0.01
V_02_max (ml • kg^-1^ • min^-1^)	46.8 (30.3-63.2)	39.8 (25.1-51.0)	< 0.01

BMI = Body mass index; HDL = High-density lipoprotein cholesterol; LDL = Low-density lipoprotein cholesterol; Non- HDL-cholesterol = Total cholesterol minus HDL-cholesterol; VO_2_max = Maximal oxygen uptake during a cycle ergometry test.

### Blood sampling

Venous fasting blood samples were drawn after 12 h of fasting from the ante-cubital vein into standard vacuum tubes and centrifuged (3000 g at 10 min) within 30 min. Plasma was immediately stored at -20°C until analysis. A standard two-hour oral glucose tolerance test with an oral glucose load of 1.75 g of glucose per kilogram bodyweight (maximum 75 g glucose) was performed. Blood samples were drawn with 30 min intervals for determination of glucose and insulin. The area under the curve (AUC) for plasma glucose (AUC_glu_) and plasma insulin (AUC_ins_), respectively, was calculated by the trapezoidal rule.

### Analyses

Serum-FGF-21 concentrations were measured with a commercial enzyme-linked immunosorbent assay (BioVendor Human FGF-21 ELISA, BioVendor, Brno, Czech Republic). Determination of FGF-21 was done on previously unthawed biobanked serum samples. FGF21 was analyzed according to the manufacturer’s instruction and measured in duplicates. According to the manufacturer, the limit of detection was 7 pg/mL, but the lowest standard was 30 pg/mL, which then in our study was set to the limit of detection (LoD). The manufacturer reported the intra-assay and inter-assay coefficients of variability (CV) to be below 5%, respectively, and with no cross-reactivity with human Fibroblast Growth Factor 19 or Fibroblast Growth Factor 23. Our inter-assay CV’s for the low (mean: 137.7 pg/mL) and high (mean: 536.3 pg/mL) controls were 7.0% and 5.5%, respectively.

Plasma-concentrations of adiponectin and leptin were measured using specific high-sensitive human enzyme linked immunosorbent assays. The adiponectin assay (Millipore, Human ADIPONECTIN RIA-kit, St Charles, Mi, USA) had intra-assay and inter-assay CV’s of 4.9% and 5.4%, respectively. Detection limits were 1 – 200 ng/mL. The leptin assay (R&D Systems, Human Leptin Immunoassay, Minneapolis, Mn, USA) had intra-assay and inter-assay CV’s of 3.4% and 1.6%, respectively, and detection limits of 7.8 – 1000 pg/mL. Glucose, triglycerides, total cholesterol, high-density lipoprotein (HDL)-cholesterol, low-density lipoprotein (LDL)-cholesterol, apolipoprotein A1 and B, respectively, were all analyzed in heparin-plasma on the Modular® *ANALYTICS* SWA, Modular P-system (Roche Diagnostics GmbH, Mannheim, Germany), using the calibrator for automated systems (CFAS) and the Roche Modular® reagents for all assays [24]. Insulin was analyzed in (heparin-plasma) determined by an electrochemiluminescence immunoassay (Elecsys insulin reagents kit; Roche Diagnostics GmbH, Mannheim, Germany) on the Modular® *ANALYTICS* SWA, Modular E170-system (Roche Diagnostics GmbH, Mannheim, Germany) [21]. Non-HDL-cholesterol was calculated as total cholesterol subtracted HDL-cholesterol.

### Statistics

All FGF21 values below the lowest standard (30 pg/mL) were set at 15 pg/mL. Mann–Whitney *U*-test was used to evaluate differences in FGF21 and metabolic parameters between genders. Differences in median levels of FGF21 in different age groups (2-yrs intervals) and pubertal stages were evaluated with Kruskal-Wallis test. To account for samples below detection, sex-specific tertiles of increasing FGF21 levels were generated. Differences between several groups were evaluated with the Kruskal-Wallis test, and differences between two groups (single or combined) were evaluated by the Mann–Whitney *U*-test. All statistical analyses were done using the statistical software IBM SPSS version 19.0 for Microsoft Windows XP (Chicago, IL).

### Ethics

The study was done in accordance with the ethical principles of the Helsinki II declaration. The COPENHAGEN Puberty Study was registered in ClinicalTrials.gov (identifier NCT01411527), and approved by the local ethics committee (reference no. KF 01 282214 and KF 11 2006–2033). All children and parents gave their informed written consent.

## Results

**Figure 1:**
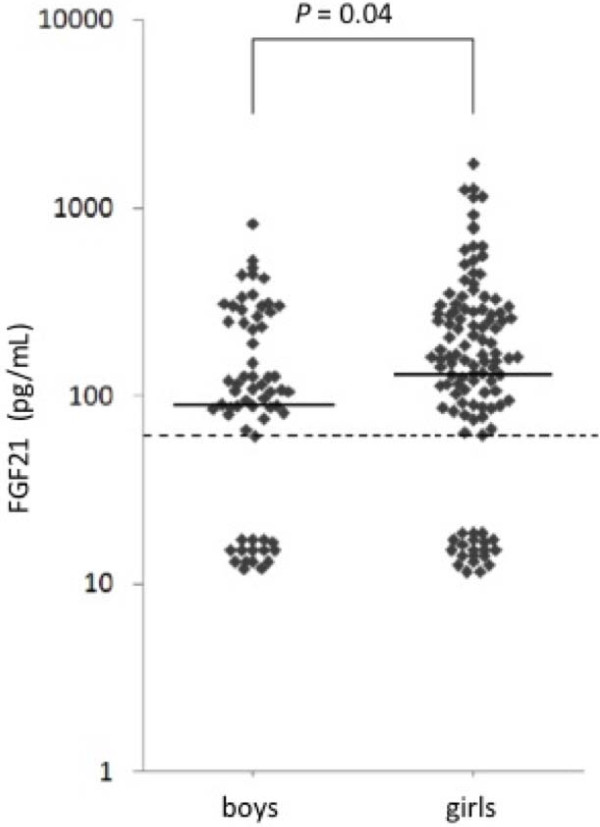
**Serum concentration of Fibroblast Growth Factor 21 (FGF21) in a group of 179 healthy, non-obese children (114 girls, 65 boys).** Each square represents the FGF21-serum concentration in a child. In 16 boys and 22 girls the FGF21-concentration was below the lowest standard of the assay; these values were set at 15 pg/mL. The solid vertical lines indicate the median values of serum-FGF21 in girls and boys, respectively. The dotted lines indicate the lowest standard of the assay. Note that the Y-axis is logarithmic.

In total, 38 study participants (16 boys, 22 girls) had FGF21 levels below the lowest standard of 30 pg/mL. The serum concentrations of FGF21 in samples above this LoD ranged from 60.6 – 1715.1 pg/mL. As shown in Figure 1, the girls had significantly higher serum-levels of FGF21 (median: 155.1 pg/mL, range < LoD - 1715.1 pg/mL) compared with the boys (median: 105.1 pg/mL, range < LoD - 818.9 pg/mL, *P* = 0.04). No significant differences were found in serum FGF21 levels between age-groups and pubertal stages in boys and girls, respectively. Neither did we find any association between baseline levels of FGF21 and glucose and insulin levels during the OGTT. Due to lack of statistical significant differences in all measured parameters between the middle and highest tertiles, these two groups were combined and compared with the lowest tertile. The lowest tertile of serum FGF21 had significantly lower triglyceride (TG) levels (median: 0.61 mmol/L, range: 0.42 – 1.77 mmol/L) compared with the combined higher tertiles (median: 0.72 mmol/L, range: 0.35 – 2.38 mmol/L, *P* = 0.01).

In sex-specific analyses, TG levels in girls were lower in the lowest FGF21-tertile (median: 0.70 mmol/L, range: 0.42 – 1.77 mmol/L) compared with the higher FGF21-tertiles (median: 0.82 mmol/l, range: 0.35 – 2.03 mmol/L), *P* = 0.03). Similar absolute differences were found for TG levels in boys between the lowest tertile (median: 0.55 mmol/L, range 0.43 – 1.41 mmol/L) and the higher tertiles of serum FGF21 (median: 0.65 mmol/L, range: 0.37 – 2.38 mmol/L), *P* = 0.11). In addition, LDL-cholesterol levels were lower in the lowest FGF21-tertile compared with the higher tertiles in girls only (*P* = 0.07).

No differences in height, weight, BMI, total body fat percentage (all p > 0.12) between the lowest FGF-21 tertile compared with the higher tertiles for girls and boys, respectively.

When the group of sixteen children and adolescents with BMI above the 85th percentile for age were compared to the large group of normal-weight children they only showed significant difference in BMI, total fat percentage, fasting insulin and leptin (p < 0.01) but no difference was found with either triglycerides, lipids, cholesterol or FGF21. These findings were consistent in a gender-specific analysis.

## Discussion

In the present study, we did not find consistent evidence in favor for a regulatory function of baseline FGF21-concentrations on glucose homeostasis. This may reflect the narrow biological range in the present sample of healthy children with normal glucose tolerance. In accordance, Gälman *et al.* found no evidence for a relationship between FGF21 levels and metabolic parameters in healthy adult subjects, [10] indicating that significant metabolic effects of FGF21 may be limited to pathological conditions associated with glucose intolerance. In addition, as FGF21 levels were only evaluated at baseline, dynamic changes in FGF21 levels during an OGTT could not be determined in the present study. One study reported that changes of FGF21 concentrations were negatively correlated with changes of glucose levels during a standard OGTT in both healthy and insulin resistant individuals [25].

Despite the lack of association with DXA-derived total body adiposity, we found positive correlations between serum-concentrations of FGF21 and triglycerides. This is consistent with the study by Tyynismaa *et al.*[26] reporting that high FGF21-levels were related to higher proportions of liver fat and higher triglycerides levels rather than to total body adiposity. Due to the cross-sectional design in the present study, the cause and effect-relationship could not be determined. However, evidence suggests that FGF21 increases in response to increasing levels of lipids, which has been hypothesized as a defense mechanism against lipotoxicity [3]. Thus, FGF21 may be a marker of elevated levels of lipids even in healthy normal-weight children. In accordance with the lack of association between FGF21 and anthropometric and biochemical parameters associated with obesity, we found no correlation between FGF21 and the adipokines leptin and adiponectin [27,28].

FGF21 levels were significantly higher in girls compared with boys, which may partly be related to the higher triglyceride levels in girls. No previous studies have addressed the possible sexual differences in FGF21-concentrations in either adults or children.

A higher number of girls are represented in the present study, which might explain why a significant difference between the metabolic parameters and FGF21 levels is seen in girls only.

The strength of our study is the large unselected sample of 179 well-characterized children. However, experimental and clinical data suggest that a certain amount of adipose tissue should be present in order for FGF21 to exert a significant glucose lowering effect [29]. Thus, evaluation of FGF21 levels in children with obesity and glucose intolerance may shed further light on the possible regulatory actions of FGF21.

## Conclusion

Fasting concentrations of FGF21 were significantly positive associated with levels of triglycerides in girls independently of adiposity and serum leptin levels. Baseline FGF21 concentrations were not associated with glucose or insulin concentrations during a two-hour OGTT. The girls showed significantly higher levels of FGF21, which may partly be explained by higher triglyceride concentrations in girls compared with boys.

## Competing interests

Anders Juul is principal investigator in scientific muliticenter trial on the effects of growth hormone on growth in short SGA children. The study receives financial support from Novo Nordisk. The remaining authors have nothing to declare.

## Authors’ contributions

AB contributed to study design, statistical analyses, data analyses and interpretation and drafted the manuscript; KS conceived the study, contributed to study design, statistical analyses, data analyses and interpretation and made important revisions of the manuscript; THJ contributed to study design, data analyses and interpretation and made important revisions of the manuscript, JWH participated in data analyses and interpretation and made substantial revisions of the manuscript; AMA participated in data analyses and interpretation and made substantial revisions of the manuscript; AJ conceived the study, contributed to study design, data analyses and interpretation and made important revisions of the manuscript. All authors read and approved the final manuscript.

## Aknowledgments

The Copenhagen Puberty Study received financial support from the Kirsten and Freddy Johansen’s foundation, the Capital Region of Denmark’s Research Fund for health research (R129-A3966), the Danish Research council (DFF-1331-00113) and the Danish Agency for Science, Technology and Innovation (09–067180).
